# Directing gaze to maximize surface color information from natural scenes

**DOI:** 10.1038/s41598-026-46685-6

**Published:** 2026-05-07

**Authors:** David H. Foster, Kinjiro Amano, Sérgio M. C. Nascimento

**Affiliations:** 1 Department of Electrical and Electronic Engineering, University of Manchester, Manchester, M13 9PL UK; 2 Physics Center of Minho and Porto Universities (CF-UM-UP), University of Minho, Braga, 4710-057 Portugal

**Keywords:** Natural scenes, gaze behavior, visual information, cone photoreceptors, color gamut, deep neural networks, Neuroscience, Psychology, Psychology

## Abstract

Natural scenes are often spatially and spectrally complex with a variety of land covers. Because of this complexity, where we look may not be guided by simple well-defined local image features or objects. Instead, gaze behavior may satisfy a more fundamental need: to reduce uncertainty about a scene’s elementary content, represented by the surface color at each point. The purpose of this study was to determine how effectively information is acquired by cone photoreceptors from scene fixations and how it relates to gaze behavior, scene variation, predictions of a deep neural network, and the number of available cone classes. Estimates of the information were computed from two sets of natural scene gaze data, one where observers searched for a target and the other where they viewed scenes freely. The information acquired in both tasks approached the maximum possible, with similar estimates from the network model. It increased with scene color diversity, quantified by color entropy, and decreased with fewer cone classes, as in some color vision deficiencies. These findings suggest that gaze is directed towards maximizing elementary scene information, which can then support more complex visual representations of scene content.

## Introduction

Our vision is sharpest at the center of gaze. Consequently, when viewing a scene, we need to move our eyes from point to point, usually several times a second, to discover more about its content. Yet what determines which points we fixate has remained problematic. The most common rationale has been the notion of physical or bottom-up salience^1,2^, that is, the presence of local image properties which by virtue of their distinctiveness^3–5^ attract an observer’s gaze. These are typically local features such as line orientation, color, and movement^6,7^, but may extend to higher-level properties representing the objects in a scene^8,9^. A notion of salience has also been intrinsic to many computational models of gaze behavior, from classical implementations with predefined local features to deep neural network models trained on fixation data^10–13^. Additional factors influencing observers’ gaze are the viewing task^14,15^, the observer’s implementation of the task^16^, and memory^17^.

When scenes are drawn from a natural environment, explaining gaze may present a greater challenge still^18^. Natural scenes are often spatially and spectrally complex, containing a variety of different types of land cover, including trees, shrubland, ferns, flowers, as well as buildings and treated surfaces^19,20^. Even over short distances, spatial variations in surface orientation may produce variations in reflected spectra, further confounded by spatial variations in the incident light^21,22^. Given this complexity, where observers look may be difficult to relate to well-defined local features and objects. Alternative rationales have been proposed that emphasize more the reduction of uncertainty about the environment and its content and the information gains made by observers^18,23,24^.

Clearly, information about scenes and its expression depend on where it is evaluated in the visual pathway. As a rule, however, processing at a higher level in the pathway cannot improve the inferences made from data obtained at a lower level^25,26^, though redundancy can be manipulated^27–30^. It is the lowest level, that of the long-, medium-, and short-wavelength-sensitive (L, M, and S) cone photoreceptors of normal trichromatic vision, that sets the limit on what information can be acquired from fixations (signals from rod photoreceptors and intrinsically photosensitive retinal ganglion cells were excluded because of their different retinal distributions and spatial and temporal resolutions^31–34^). This approach depends only on the spectral properties of the light reflected at each point at a particular time, not on the local features or objects they might delineate^35–37^, or their variation over time^38^, or their semantic content^39^, all of which entail assumptions about post-receptor processing of multiple cone signals. Some of these issues have been addressed elsewhere, for example, in analyzing the cone mosaic^40–43^ and the postreceptor coding of images of natural scenes and objects^30,44,45^.

Information is taken as Shannon’s mutual information^25^, that is, the reduction in uncertainty about one random variable – the reflectance at each point in a sample of points from the scene – given knowledge of another random variable – the resulting cone response. This application to data transmission should be distinguished from its use in model comparison, especially in assessing how well deep neural network models predict eye fixation patterns^13,46^. In general, information is measured in binary digits or bits, where one bit corresponds to a gain or loss of information by a factor of two. Its logarithmic inverse may be interpreted as the effective number of surfaces in a scene that can be distinguished by virtue of their reflecting properties^47^. It necessarily depends on the composition of the scene as a whole, the visual context^48^, rather than on individual surfaces taken one at a time.

The purpose of this study was to determine how effectively information is acquired by cones from scene fixations, and how it relates to gaze behavior, scene variation, network model predictions, and number of cone classes. It is not concerned with the prediction of specific fixation locations or the saccades between them^49–52^.

**Fig. 1:**
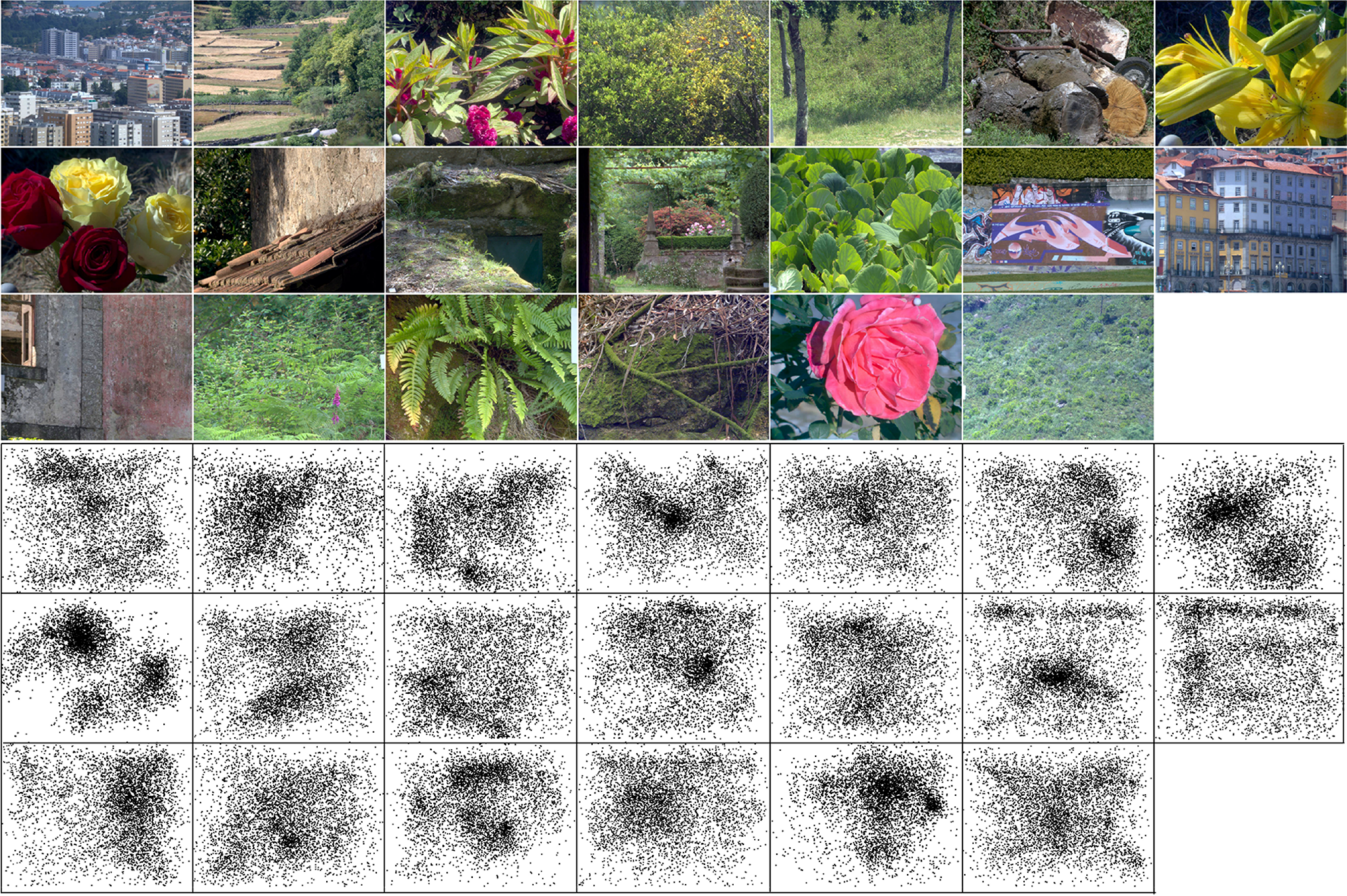
Scenes and observer fixation locations in a target search task^53,65^. The color images in the upper three rows are standard red-green-blue sRGB images^89^ of 20 natural scenes rendered from hyperspectral radiance images. The pooled distributions in the lower three rows are each a random sample of about 5600 fixation locations from the corresponding scene viewed by 7 observers over 260 trials each lasting 1 s.

**Fig. 2:**
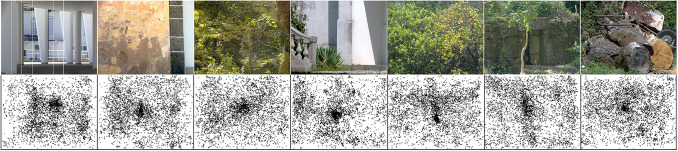
Scenes and observer fixation locations in a free-viewing task^54,55^. The pooled distributions in the lower row are each a random sample of about 5600 locations in the corresponding scene viewed by 5 observers continuously for 5 min. Other details as for Fig. 1.

Estimates of the information from observed fixations were computed in two different tasks with two sets of gaze data from natural scenes. The main set, illustrated in Fig. 1, was from a laboratory experiment in which observers searched images of scenes for a small, randomly embedded target^53^. The secondary set, illustrated in Fig. 2, was also from a laboratory experiment in which observers freely viewed images of scenes^54,55^, and, for comparison, in which two of the observers viewed the same scenes outdoors^54,55^. Images were rendered from hyperspectral data. Although free viewing is not always well defined^16^, it can offer a useful comparison with an explicit search task.

It was found that observers’ gaze distributions delivered information that approached the maximum possible for most scenes, with similar estimates from the chosen network model. Information increased with scene color diversity and decreased with fewer cone classes. It is suggested that gaze is directed towards maximizing elementary scene information, as a precursor to more complex visual representations.

Preliminary reports of this work were presented to the 27th Biennial Meeting of the International Colour Vision Society, Ljubljana, Slovenia, 2024, and to the Optica Fall Vision Meeting^56^, York, UK, 2024.

## Results and comment

For technical details, see the Methods section for the estimation of the information acquired from observer fixations of scenes and also the estimation of baseline and maximum values with other sampling regimes. Unless otherwise indicated, results are reported for laboratory measurements.

### Target search and free viewing

**Fig. 3:**
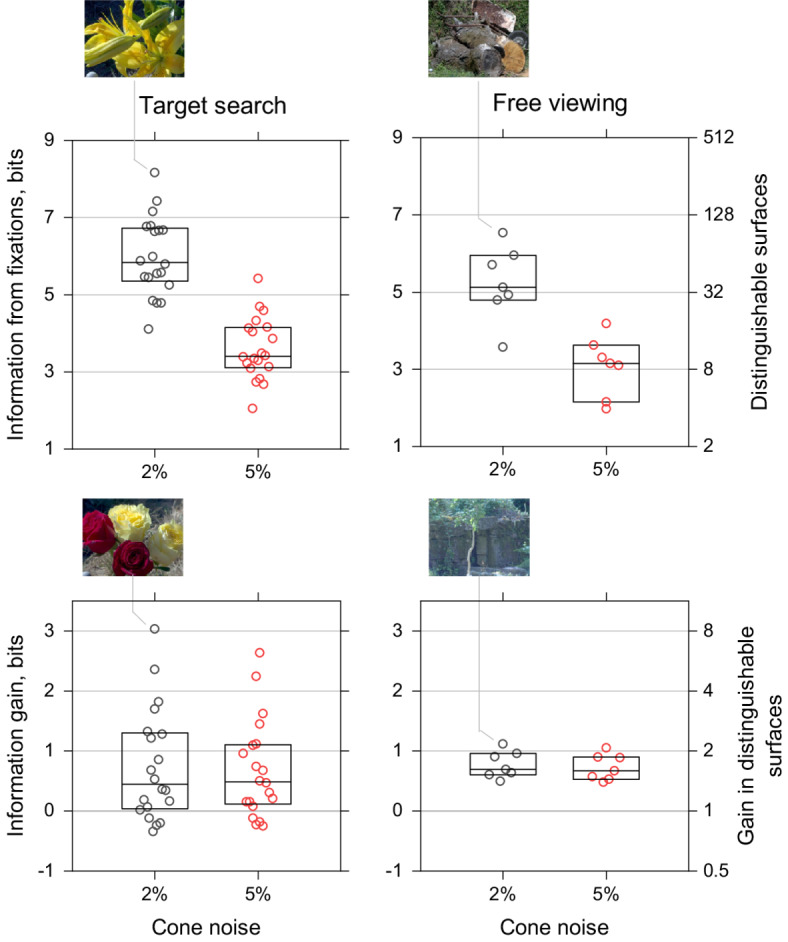
Information from observer fixations of scenes for two assumed levels of cone noise. Each data point in each column scatter plot is for an individual scene. The upper left panel shows estimated mutual information from target search and the upper right panel from free viewing. The lower panels show the corresponding plots of the estimated gain in mutual information with respect to sampling points from each scene uniformly at random. The right-hand scales show the effective numbers of distinguishable surfaces (see Methods). The boxes superimposed on the data points mark median and quartile values over 20 scenes for target search (Fig. 1) and over 7 scenes for free viewing (Fig. 2). Data points have been jittered horizontally for clarity. The thumbnail images show scenes with the largest information estimates with 2% cone noise (the scenes were the same with 5% cone noise). Notice the different vertical scales for mutual information and mutual information gain.

The upper panels in Fig. 3 show information estimates from fixations in target search and free viewing for two levels of cone phototransduction noise assumed to limit sensory performance. Each data point in the column scatter plots represents an estimate from one of the scenes in Figs. 1 and 2. In target search, the median estimate across the 20 scenes with 2% cone noise was about 5.8 bits (CI 5.4 to 6.7 bits), which corresponds to an effective number of distinguishable surfaces of about 57 (values depend on both image and receptor spaces). With 5% cone noise, the median estimate fell to about 3.4 bits (CI 3.2 to 4.0 bits), and with 10% cone noise (not shown), it fell to 2.0 bits (CI 1.8 to 2.5 bits). In free viewing, the median estimate across the 7 scenes with 2% cone noise was about 5.1 bits (CI 4.8 to 6.0 bits). With 5% cone noise, it fell to about 3.1 bits (CI 2.2 to 3.6 bits), and with 10% cone noise, to about 2.1 bits (CI 1.0 to 2.3 bits).

The lower panels in Fig. 3 show the corresponding estimates of the information gain with respect to points sampled uniformly at random from each scene. Random sampling provides a baseline measure of the information available independent of observer measurements, in other words, what would be expected by chance.

In target search, at least 15 scenes out of the 20 tested yielded positive gains, with median values of 0.5 bits (CI 0.1 to 1.3 bits) for 2% cone noise and 0.5 bits (CI 0.2 to 1.0 bits) for 5% cone noise. The distribution of gains was positively skewed and the respective 90th percentiles were 2.1 bits (CI 1.5 to 3.0 bits) and 1.9 bits (CI 1.1 to 2.6 bits). In free viewing, all 7 scenes yielded positive gains, with respective median values of 0.7 bits (CI 0.6 to 1.0 bits) and 0.7 bits (CI 0.5 to 0.9 bits). The sample size was too small for percentile estimates. Given the predictable effects of increases in cone noise, information estimates are subsequently reported only for a representative level of 2% cone noise.

For the 7 scenes viewed freely outdoors by two observers, the median paired difference between outdoor and indoor information estimates was 0.0 bits (CI − 0.7 to 1.1 bits) with 2% cone noise.

These results are considered in more detail in the following sections.

### Maximum information

To determine the effectiveness of fixations in acquiring information, estimates were made of the maximum values theoretically possible for each scene. These maxima were derived by sampling from a subset of locations chosen optimally for each scene but subject individually to shifts modeling fixational eye movements^57–59^, the small movements that the eye undergoes during fixations. Shifts were assumed to be random in both direction and amplitude with amplitude values distributed either uniformly up to 1 deg of visual angle^58–60^ or according to an empirical unimodal distribution^61^.

The median estimate of the maximum information over the 20 scenes in target search was about 6.8 bits with uniform amplitude shifts and about 7.2 bits with unimodal amplitude shifts. The median estimate of 5.8 bits from observer fixations of the 20 scenes reported in the previous section was, respectively, 86% and 81% of these estimated maxima. The corresponding estimate of 5.1 bits from observer fixations of the 7 scenes in free-viewing was similar, respectively 87% and 85% of the estimated maxima.

### Short presentations

Both observer tasks involved repeated or prolonged viewing of scenes, and it was not obvious^8,18^ whether similar information estimates would be obtained with short presentations. To resolve this issue, the analysis in target search was restricted to fixations from the first 1-s trial with each scene. The median information estimate across scenes was 5.8 bits (CI 5.3 to 6.2 bits), indistinguishable from the estimate of 5.8 bits with all 260 trials for each scene reported earlier. The analysis in free viewing was restricted to fixations from the first 3 s viewing with each scene, as shorter intervals contained too few fixations. The median across scenes was 6.0 bits (CI 5.5 to 7.5 bits), reliably larger than the estimate of 5.1 bits with the full 5 min exposure for each scene but indistinguishable from the estimate in target search.

### Central bias

With scenes or images of limited spatial extent, observers may look more towards the center of the scene than elsewhere. This central viewing bias^62–64^ could contribute a common component to the information from fixations in each scene, and offers the possibility of acting as a more relevant baseline distribution instead of the uniform distribution used for information gain in Fig. 3, lower panels.

To test this notion, a bias distribution was constructed for each scene by replacing the sample of observer fixations by a sample of the same size drawn evenly from the fixations in all the other scenes in the set (thus 19 out of 20 scenes in target search and 6 out of 7 scenes in free viewing). Except for the exclusion of the current scene, the construction was the same as in a previous investigation into how local scene color accounts for fixations^65^. If a central viewing bias contributed as suggested, then for each scene the information gain with respect to it should be less than (or at least not exceed) that with respect to uniform sampling, and furthermore, over all scenes, the variance of the information gain should be less than with respect to uniform sampling.

**Fig. 4:**
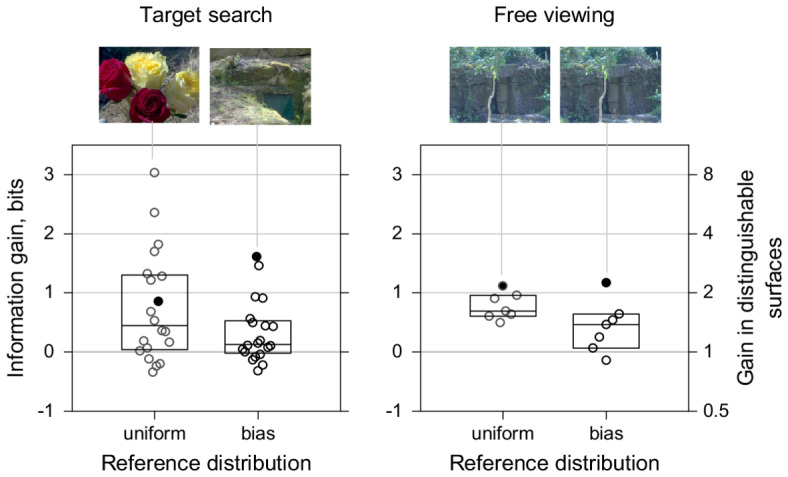
Information gain from fixations with respect to two different baseline distributions. In each panel, the estimated gain in the left column scatter plot, reproduced from Fig. 3, is for a uniform baseline distribution and in the right column scatter plot for a central bias distribution estimated from the other scenes. The filled symbols identify scenes giving anomalous effects. Cone noise was assumed to be 2%. Other details as for Fig. 3.

Figure 4 shows the estimated information gain with respect to uniform sampling, reproduced from Fig. 3 for comparison, and the corresponding gain with respect to the bias distribution. Data in the left panel are from target search with 20 scenes and in the right panel from free viewing with 7 scenes.

The median (and mean) estimated gain decreased with both sets of scenes, as required. The variance also decreased with the 20 target search scenes but not with the 7 free viewing scenes, where it increased by a factor of 3.6 (CI 1.0 to 11.4). Individual effects with some scenes were revealing. Thus with one scene in target search (filled symbols, left panel in Fig. 4), the gain increased from 0.9 bits to 1.6 bits, due to the baseline level of information from the bias distribution falling 0.7 bits below chance with uniform sampling. With another scene in free viewing (filled symbols, right panel in Fig. 4), the gain remained almost the same at around 1.1 bits but it contributed to an increased variance of the gain because of the decrease in the median (and mean) of the bias information. Both of these effects are incompatible with the bias distribution acting as a baseline for gain.

### Network model predictions

A natural question is whether fixation locations predicted by a deep neural network model deliver information estimates similar to those from observers. The model in question was the deep neural network model DeepGaze IIE^66^, whose particular relevance is summarized in the Methods section. Its use here was intended to be illustrative rather than necessarily representative of network models as a whole.

The median estimate of the information from fixations predicted by DeepGaze IIE with the 20 scenes in Fig. 1 was 6.1 bits (CI 5.8 to 6.5 bits), a little larger than the estimate of 5.8 bits from observer fixations in the search task with the same scenes. The median prediction for the 7 scenes in Fig. 2 (two scenes in common with Fig. 1) was 4.7 bits (CI 4.4 to 6.1 bits), a little smaller than the estimate of 5.1 bits from observer fixations in the free-viewing task with those scenes.

**Fig. 5:**
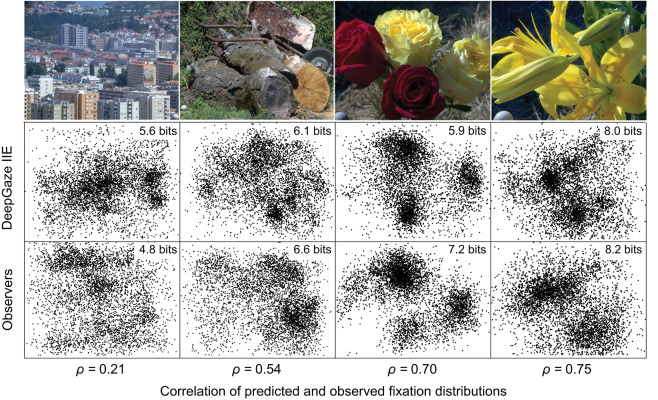
Predicted and observed fixation distributions for four scenes from Fig. 1 and their correlation coefficients *ρ*. The middle row shows predictions of a deep neural network model DeepGaze IIE^66^ and the bottom row shows observer fixations (reproduced from Fig. 1). Mutual information estimates from fixations are shown in the top right corner of each distribution. Other details as for Fig. 1.

The signed differences between information estimates from observer fixations and DeepGaze IIE predictions varied from − 0.83 bits to 1.25 bits across the 20 scenes in target search. This variation is illustrated for four scenes in Fig. 5. The middle row shows predicted fixation locations for four scenes and their information estimates and the bottom row shows corresponding data for observer fixation locations (reproduced from Fig. 1). The columns are ordered by ascending correlation between observed and predicted locations quantized^65^ for calculation into 17 × 13 = 221 bins. Differences in fixation distributions were only weakly linked to information differences. Over all 20 scenes, the correlation coefficient was 0.38 (CI − 0.07 to 0.67). This issue is considered in the Discussion.

### Variation with scenes

The data in Fig. 3 and the examples in Fig. 5 demonstrate how the amount of information from fixations varies across scenes. To test a natural predictor of this variation, scenes were characterized by a measure of their color diversity^67–69^ that takes into consideration the frequency of occurrence of individual surface colors, in other words, their color entropy^67,70^.

**Fig. 6:**
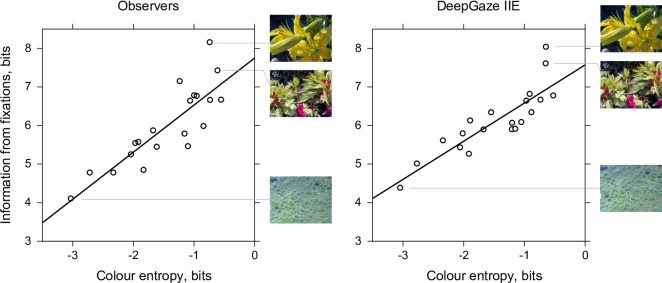
Variation of information with scene color diversity. Estimates of the mutual information from observer fixations and from a deep neural network model DeepGaze IIE^66^ are plotted against the differential entropy of surface colors in the 20 scenes of Fig. 1. Cone noise was assumed to be 2%. The slanting lines show linear regression fits to the data in each panel. Overlapping data points have been displaced horizontally.

Figure 6 shows the information from fixations plotted against color entropy for the 20 scenes of Fig. 1. Data in the left panel are for observer fixations from target search and in the right panel from fixation locations predicted by DeepGaze IIE. The slanting lines are linear regression fits to the data in each panel. The adjusted proportion *R*^2^ of variance accounted for was 71% and 73%, respectively, for observers and DeepGaze IIE. The thumbnail images show two scenes with the largest information estimates and color entropy and one scene with the smallest information estimate and entropy.

### Fewer cone classes

Reducing the number of cone classes assumed to be available to observers, as in some color vision deficiencies, should reduce the amount of information from scenes in an orderly way. This theoretical manipulation does not model all inherited color vision deficiencies, which may also involve shifts in cone pigment spectra^71,72^, but reveals the effect of the reduction in dimensionality of the cone representation.

Table 1 shows the resulting median information estimates and effective numbers of distinguishable surfaces across the 20 scenes of Fig. 1, given the fixation locations chosen by observers with normal trichromatic vision in target search. Expressed as a proportion of the information with all three cone classes, the largest residual information of 5.5 bits was 94%, mediated by L and S cones, and the smallest residual information of 2.3 bits was 40%, mediated by S cones alone. Losses in the effective number of distinguishable surfaces were proportionally greater, as this number scales with the exponent of the information.

The information from fixations with L or M cones alone may be regarded as a proxy for a response to the luminance or achromatic content of scenes. The residual information was 79% and 76%, respectively, with each of these two cone classes, a finding which has parallels in scene analyses without reference to fixational behavior^73^.

**Table 1:** Information from scene fixations by observers with different combinations of long-, medium-, and short-wavelength-sensitive (L, M, and S) cones.

Cone classes	Information from fixations, bits	Distinguishable surfaces
L, M, S	5.8 (CI 5.4 to 6.7)	57
L, S	5.5	44
M, S	5.2	37
L, M	5.3	40
L	4.6	24
M	4.5	22
S	2.3	5

Numerical entries (rounded to 2 significant figures) show median estimates of the mutual information across the 20 scenes of Fig. 1 and the effective numbers of distinguishable surfaces (see Methods). Cone noise was assumed to be 2%. Reference data in the first row for normal trichromatic vision were taken from the data summarized in Fig. 3.

## Discussion

Directing our gaze from point to point in a scene informs us about its distinct reflecting elements. The amount of information acquired varies with the scene, and for the twenty natural scenes tested here, it was equivalent to distinguishing effectively between about 20 and 300 surfaces in each scene. Around 70% of this variation could be accounted for by the diversity of surface colors, captured by their color entropy. Corresponding information estimates from fixations predicted by a deep neural network saliency model were similar, though the predicted fixation distributions sometimes differed from those by observers. As expected, information decreased with the number of cone classes assumed to be available. These findings and their implications are examined in what follows.

The observer’s task has long been understood to strongly influence gaze behavior^74,75^. Target search is unambiguous, whereas free viewing is inherently less well defined and has been argued to allow observers to adopt their own strategies, equivalent to performing different, unknown tasks^16^. Yet the median information from fixations in the free viewing task was closely similar to that in the target search task. One reason may have been that in both tasks observers had to explore each scene: in free viewing to prepare for an unknown question about the scene and in target search to determine whether or not the target was present. Neither task required observers to distribute their fixations uniformly. For the two observers who viewed scenes indoors and outdoors, outdoor viewing offered no advantage^76^, but it is unclear whether this would hold more generally.

Unsurprisingly, the median information acquired from scenes decreased as the number of cone classes assumed to mediate responses decreased, owing to the poorer sampling of the reflected spectrum at each fixated point. Moreover, for a given number of cone classes, the variation in the information with different combinations of cone classes is consistent with the spacing of their spectral peaks and the properties of the image spectra at short wavelengths^73^. For example, with two cone classes, the L and M cones have the smallest spectral spacing and therefore give the least effective spectral sampling of all three pairings of L, M, and S cones. With only one cone class, S cones sample the spectrum where reflected radiance is usually lower and noise effects are greater.

The loss of either M or L cones alone had the smallest impact on the information acquired, because responses could still be mediated by the remaining L and S cones or M and S cones. The resulting median information estimates were at least 90% of the estimate with all three cone classes. It is noteworthy that in practice observers with the more common inherited color-vision deficiencies – anomalous trichromacy or dichromacy – who therefore retain at least both L and S cones or both M and S cones, have been found to distribute their fixations over artworks and natural scene images in a way little different from normal controls, although taking longer to do so^77^.

Reductions in the number of cone classes also give an insight into the balance of cues to scene content. Information from fixations with L or M cones alone constituted more than about 75% of the median information available with all three cone classes. As proxies for luminance or achromatic variation, they provide the majority of the information from scenes, as has been shown previously without reference to fixation distributions^21,22,73,78^. Nevertheless, chromatic cues contributed substantial additional information.

Two potential experimental confounds were tested in the course of the analysis. One was the influence of repeated or prolonged viewing of scenes. Expectations based on previously published reports are complicated by the variety of viewing environments and tasks, with evidence offered for both an effect of repetition^79,80^ and little or no effect^81,82^ with naturalistic or complex scenes. In the target search task, information estimates from fixations made in only the first 1 s trial with each scene were no different from those made in all 260 trials. This may have been due to the uniform distribution of target locations over each scene and observers not receiving feedback on performance, so that changing gaze strategy offered no obvious benefit. By contrast, in the free viewing task, the long observation period may have reduced gaze activity, biasing the distribution of fixations and reliably diminishing the information acquired with respect to the first 3 s of viewing.

The other potential experimental confound was a common contribution across scenes to information from a central viewing bias in observer fixations^65^, whether arising from scene composition^64,83^ or general gaze behavior^3,62–64,84,85^. When used as a baseline, the bias distribution reduced the mean and median gains over scenes, consistent with its putative role, but at the cost of producing anomalies with individual scenes. Most notably, the information it estimated with one scene fell well below chance, so that in advance of an observer’s measurements, the prediction would have been poorer than by sampling scene points uniformly at random^86^. This effect can arise when color diversity attracts fixations away from the center of a scene, with the result that for sufficiently mismatched sampling, the information falls below that from a uniform distribution, which treats each part of the scene equally.

There are several limitations of this analysis. First, estimates of the maximum possible information from each scene provided only lower bounds, in the sense that further optimization might have produced higher values, albeit unreliably so given the size of the estimated confidence intervals. More importantly, the lowering of the bounds on estimates because of fixational movements about the optimal locations depends on how cone signals from those movements are exploited. As noted earlier, recoding cannot increase information, but adding side information^25,26^ can have that effect, for example, with positional data from low and high spatial frequencies^57,61^ acting to reduce uncertainty about surface location. On the other hand, in practice, fixational movements may have only a small effect on perceived color^87^.

Second, for various reasons, saliency models do not always extend well to real-world scenes^88^. The fact that the network model DeepGaze IIE^66^ delivered information estimates similar to those from observer fixations may have been due to its capacity to accommodate unseen datasets. Other network models may perform differently, and an exhaustive evaluation would be needed to settle the issue.

Third and last, the information estimates reported here apply to scenes in their entirety. The interpretation of information as the effective number of surfaces in a scene that can be distinguished by virtue of their reflecting properties makes sense only for scenes as a whole, not for surfaces in isolation. Although the analysis does not predict fixation locations, it does identify distributions of locations yielding the maximum information theoretically possible. These locations need not be unique.

The simplest interpretation of the present findings is that observers’ gaze is directed towards maximizing information about elementary surface color in natural scenes. Of course, other interpretations are available based on more complex visual representations of scene content, including local features and objects. But they all rely on information acquired at the level of the cones, which ultimately constrain what can be inferred.

## Methods

The following sections set out the procedure for estimating the information acquired by cones from scene fixation data. Computations were performed in the MATLAB computing environment (Version R2024b, The MathWorks, Inc., Natick, MA). Scene and image dimensions, gaze accuracy, and fixation locations are expressed mainly in terms of visual angle but where relevant in pixels. Some of the material is abstracted from previous reports^47,53,73^.

### Images and gaze data from target search

The main set of gaze data was taken from a laboratory search experiment^53^ with 20 hyperspectral images of natural scenes, illustrated in Fig. 1, upper three rows, as sRGB^89^ color images. The scenes were natural in the sense of being part of everyday outdoor environments with a variety of undeveloped and developed land cover^19,20^, unlike laboratory and virtual constructs. The hyperspectral images were recorded as reflectance images and were converted^90^ to radiance images under an average daylight of correlated color temperature 6500 K^91^. Each image had dimensions approximately 1344 × 1024 pixels and a spectral range 400–720 nm sampled at 10-nm intervals, giving 33 wavelength values at each pixel. The point spread function of the hyperspectral camera was close to Gaussian with standard deviation (SD) of about 1.3 pixels. For access to the image dataset, see the data availability section.

Observers viewed the images at full spatial resolution on the screen of a calibrated color monitor, where they subtended approximately 17 × 13 deg at the eye at a viewing distance of 1 m (so 1 pixel subtended approximately 0.76 arcmin). The hyperspectral images were rendered on the monitor screen in the usual way^53,92^. The target was a small, shaded, gray sphere subtending 0.25 deg embedded in the scene and matched in mean luminance to its local surround to minimize accidental chromatic or luminance contrast cues to detection^53^, Fig. 1. The target appeared randomly in half of the trials at one of 130 possible locations, defined by an imaginary 13 × 10 grid. The observer’s task in each trial was to signal whether the target was present. Scenes were viewed for 1 s in each trial but were presented repeatedly in successive trials, of which there were 260 in all for each scene. Differences between information estimates with single and repeated presentations are considered in the Results section. Despite the low contrast and small size of the target (less than 2% of the image width), observers’ detection performance was well above chance, with a mean discrimination index *d'* of 1.2^53^.

Eye movements were recorded with an infra-red video eye-tracker (High Speed Video Eyetracker Toolbox mk2, Cambridge Research Systems Ltd., Kent, UK), with temporal sampling frequency 250 Hz. At the start, middle, and end of each subblock of 65 trials, the eye-tracker was calibrated against 20 calibration targets displayed in a 5 × 4 grid on the screen (these fixation data were excluded from the analysis). An affine transformation was used to transform the experimental gaze locations in each subblock to correct the screen coordinates. The root-mean-square difference between each of the twenty calibration targets and the observer’s corresponding gaze location was approximately 16 arcmin (SD 4 arcmin), evaluated over observers and scenes. The lower three rows of Fig. 1 show recorded fixation locations in black corresponding to the color images in the upper three rows.

Seven observers (4 female, 3 male; aged 21–31 years), including coauthor KA, participated in the experiment. All had normal visual acuity and normal color vision and all gave their informed consent. The experimental procedure was carried out in accordance with the relevant guidelines and regulations of the University of Manchester Committee on the Ethics of Research on Human Beings, which approved all the experimental protocols (Ref. No. 08104). Further details are available in two articles^53,65^.

### Images and gaze data from free viewing

The secondary set of gaze data was taken from a free-viewing experiment^54,55^ with 7 natural scenes, illustrated in Fig. 2, upper row, viewed either as hyperspectral images in the laboratory or, for a subset of observers, as the original scenes outdoors. For each of these scenes, the hyperspectral images were acquired first, and then outdoor gaze data recorded from observers at the same location. Laboratory gaze data were recorded separately. The physical characteristics of the hyperspectral images were the same as in the main target search experiment, and two of the images served in both experiments.

In the laboratory measurements, the images were displayed on a monitor screen in a similar way to the target search experiment except that no target was introduced. For both laboratory and outdoor measurements, observers freely viewed each scene for 5 min knowing that they would be asked a randomly chosen question about the content of the scene (e.g., how many tree trunks were present).

Eye movements were recorded with an infra-red video eye-tracker (iView, SensoMotoric Instruments GmbH, ver. 3.01, Germany), with temporal sampling frequency of 50 Hz. At the beginning of the 5-min viewing period, the eye-tracker was calibrated against a 3 × 3 grid of 9 small gray calibration targets placed in the scene. A fixation centering check was performed every minute and at the end of the viewing period, the calibration of the eye-tracker was checked (these fixation data were excluded from the analysis). Typical gaze location accuracy was 0.5–1 deg visual angle. The calibrations were preserved with the seven scenes used in this analysis, but not with three other scenes, whose data were therefore discarded. Because of the lower sampling frequency, identification of candidate fixations was less reliable than in the main experiment. The lower row of Fig. 2 shows recorded fixation locations in black corresponding to the color images in the upper row.

For the laboratory measurements, five observers (male, aged 24–59 years), including coauthors KA and DHF, participated in the experiment. All had normal or corrected-to-normal visual acuity and normal color vision. For the outdoor measurements, only two of the five observers (KA and DHF) participated. All observers gave their informed consent. The experimental procedure was carried out in accordance with the current guidelines of the Research Ethics Committee of the University of Minho to the Color Science Laboratory (CEICVS 052/2021). Further details are available in the cited documents^54,55^.

### Fixation classification

**Fig. 7:**
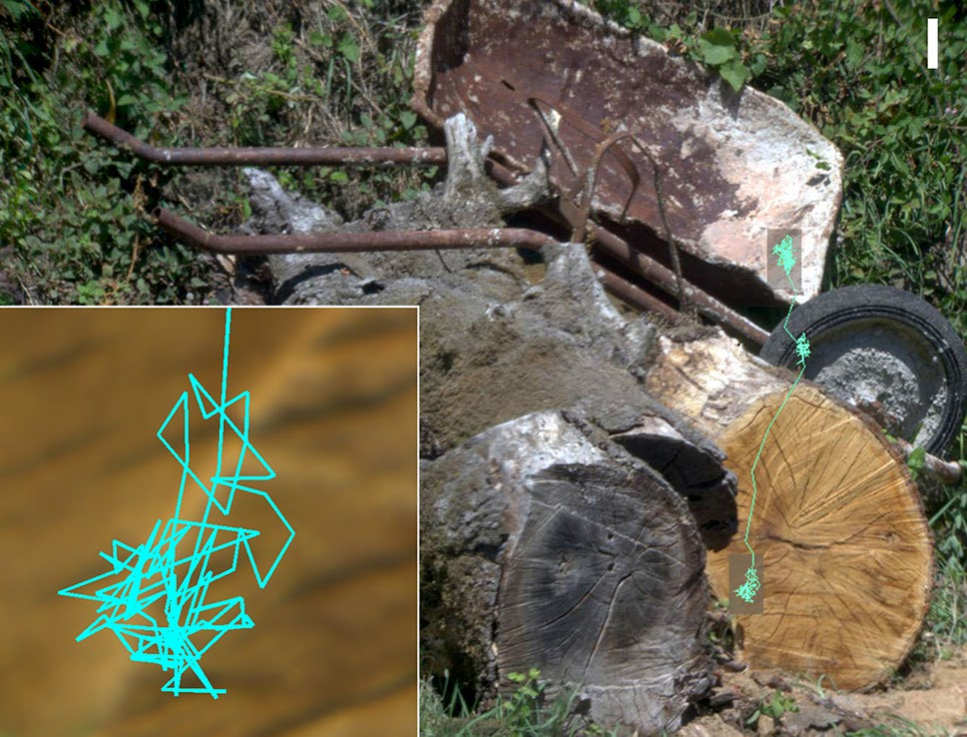
Eye-movement trace showing fixation clusters and saccades between them. The inset at the bottom left is an enlarged copy of the fixational movements at the bottom of the trace in the main image, which has been darkened locally for clarity. The vertical scale bar at the top right marks 1 deg visual angle. Adapted from data for Fig. 1 in Ref. 94.

Although eye movements divide broadly into fixations and rapid gaze shifts or saccades, the eye continues to make small movements during fixations with amplitudes up to 1 deg of visual angle^58–60^, so that each fixation represents a cluster of closely spaced locations, as illustrated in Fig. 7.

These fixation locations were extracted from both the main and secondary sets of unsmoothed^53,93^ gaze data by a nonparametric classification method^94^. It was fully automatic, requiring neither assumptions about thresholds for speed, acceleration, duration, and stability, nor expert judgment. The method was mainly speed-based, but for each observer and scene, an optimum speed threshold was derived automatically with Tibshirani, Walther, and Hastie’s gap statistic^95^. An optimum duration threshold was also derived automatically by this method to allow for the effects of instrumental noise.

### Eye tracker errors

Instrumental noise arising in the eye tracker data was accommodated by the nonparametric classification method but any systematic errors remained, with, as noted earlier, magnitudes of the order of 0.3 deg visual angle in target search and 0.5–1 deg in free viewing. Their effect on information estimates was assessed by introducing uniform random shifts of these magnitudes into the recorded fixation data for each scene. Paired comparison tests revealed no reliable effect: in target search the median difference was 0.03 bits (CI − 0.03 to 0.09 bits) and in free viewing it was − 0.07 bits (CI − 0.21 to 0.16 bits).

### Network model predictions

To provide a comparison with observer performance, information estimates were obtained from fixation locations predicted for each scene by a deep neural network saliency model DeepGaze IIE^66^, designed to give confidence in its predictions with unseen datasets. It was based on an ensemble approach: multiple different pretrained network models were combined in a principled manner, followed by other operations, including the introduction of a center bias, to generate an output consisting of a probability density function of predicted fixation locations^13,66^. The number sampled from the density function for each scene was the same as the number observed experimentally.

### Cone responses

Hyperspectral radiance images were converted routinely^90^ into L, M, and S cone excitations with the Stockman and Sharpe 2° cone spectral sensitivities, lens, and macular pigment data^96^. Cone signal processing was assumed to be limited by phototransduction noise^42,97^, which varied linearly with background level^98^. The noise distribution was modeled as an additive Gaussian process whose SD at each scene point was specified relative to the mean cone excitation over the scene (which may be approximated by the mean over successive fixated areas^55^). The coefficient of proportionality, the Weber fraction, was guided by Stiles’ psychophysical increment threshold measurements^99^, which yielded Weber fractions of 1.8% 1.9%, and 8.7% for L, M, and S cones, respectively. For brevity, representative values of the relative SD are reported just for L cones, with values for M and S cones scaled appropriately^100^. Results were derived for relative SDs of 2%, 5%, and 10%. Modeling noise with a Gaussian distribution was not critical: a uniform distribution with the same differential entropy (see Information estimates) produced closely similar information estimates. All spectral computations were carried out with the same spectral range and resolution as the hyperspectral images.

### Information estimates

For a given set of fixation locations in a scene, information was calculated between the radiance spectra at those locations and the resulting cone responses. As elsewhere^46,50^, the locations of fixations were assumed to be due to a spatially random process^101^, governed among other factors by the content of the scene and observer knowledge. The radiance spectrum at each point in a sample of points was accordingly treated as an instance **u** of a 33-dimensional continuous random variable, **U** say, with an underlying probability density function *g*(**u**) defined on the space of radiance spectra (not to be confused with the radiance spectrum itself). The uncertainty in **U** was quantified by the differential entropy^25^ *h*(**U**) defined by

1 $$h(\mathbf{U}) = - \int {g(\mathbf{u})\log \,g({\mathbf{u}}){\,\rm d} \mathbf{u}.}$$

The corresponding triplet of L, M, and S cone responses together with phototransduction noise was treated as an instance **v** of a 3-dimensional continuous random variable, **V** say, with an underlying probability density function *g*(**v**). The uncertainty in **V** was similarly quantified by the differential entropy *h*(**V**) defined by

2 $$h(\mathbf{V}) = - \int {g(\mathbf{v})\log \,g({\mathbf{v}}) {\, \rm d} \mathbf{v}.}$$

With logarithms to the base 2, differential entropy is measured in bits. The reduction of uncertainty in **U** due to knowledge of **V** is the mutual information^25^, written *I*(**U**; **V**), and is defined in terms of *h*(**U**) and the conditional differential entropy *h***(U** | **V**) by.

3 $$I\left( {{\mathbf{U}};{\mathbf{V}}} \right)=h\left( {\mathbf{U}} \right) - h\left( {{\mathbf{U}}|{\mathbf{V}}} \right).$$

It is also measured in bits. Mutual information can instead be defined in terms of the Kullback–Leibler distance between probability distributions^25^, though in this context the difference of entropies (3) is more easily related to the transmission of information^102^.

Numerical estimates of the differential entropy (1) and (2) and mutual information (3) were obtained with an offset version^103^ of the Kozachenko-Leonenko *k*th-nearest-neighbor estimator^104,105^, which converges relatively rapidly and accurately with increasing sample size^103,106^.

It is sometimes useful, as in this study, to exploit the fact that the logarithmic inverse of the mutual information gives the effective number of surfaces in a scene or image that can be distinguished by their colors. These numbers are domain dependent. For reasons to do with the dimensionality of hyperspectral images and trichromatic cone responses, estimates may differ from those obtained when images and responses are both represented in the same trichromatic spaces.

### Uniform sampling in scenes

The preceding section describes samples determined empirically by scene fixations, but it also applies to theoretical sampling regimes, in particular, when points are chosen randomly according to a spatially uniform distribution. Each sample of a given size then has the same probability of occurring^107^. In that case, the quantity in (3), the mutual information, provides a baseline measure of scene information independent of observer measurements, namely the level expected by chance. The difference between the mutual information from fixations and the mutual information from uniform random sampling is referred to as the information gain (other formulations are used in some applications^46^). Intuitively, the mutual information from fixations should exceed the mutual information from uniform random sampling (or at least not fall below it), that is, the information gain should be non-negative, except for sampling variance and cone noise. An alternative baseline measure of scene information is considered in the Results section.

Additionally, because uniform random sampling treats points equally, the quantity in (2), the entropy of colors in a scene^67^, gives a frequency-weighted measure of color diversity (encompassing both chromatic and achromatic attributes) expressed in the space of cone responses. Its logarithmic inverse gives the effective gamut volume of the surface colors, which, in the limit where colors are equally likely, reaches the classical gamut volume^47^. Of the alternative measures of color diversity^67–69^, including gamut volume and variance, entropy has the advantage that both it and mutual information are defined in terms of probability density functions on the same spaces.

### Maximum information

An estimate of the maximum information theoretically possible for a scene for a given sample size was derived by approximately equalizing the frequencies of occurrence of surface colors and then sampling uniformly from that set (as distinct from sampling from the original scene by fixations).

More precisely, with notation adapted from a previous analysis^73^, let *l*_*i*_(*λ*) denote the spectral radiance at wavelength *λ* at an arbitrary scene location (*x*, *y*)_*i*_, indexed by *i*, and let {*l*_*i*_} denote the set of all such spectra from the scene. The required subset {*l*_(*k*)_} of {*l*_*i*_}, indexed by *k*, was then obtained by thinning^108^, that is, by removing spectra *l*_*r*_ from {*l*_*i*_} within a distance *d* of other spectra *l*_*s*_ as *d* was progressively increased up to a limit that provided enough spectra for a specified sample size and for which stable information estimates were available^106^. The distance *d* between two spectra was defined by the sup metric, *d*(*l*_*r*_, *l*_*s*_) = sup_*λ*_(|*l*_*r*_(*λ*) – *l*_*s*_(*λ*)|). This estimate of the maximum is approximate, since the optimal subset need not be unique, and depends also on the definition of the distance *d* and the limiting sample size.

There is, however, another source of uncertainty that needs to be included. By construction, the foregoing optimal locations (*x*, *y*)_*i*_ were specified to within 1 pixel in images of size approximately 1344 × 1024 pixels. This precision does not take into account fixational eye movements mentioned earlier, where amplitudes of 1 deg of visual angle^58–60^ were equivalent to 79 pixels in the experiments. These fixational shifts were treated as random variables and incorporated into estimates of the maximum information in two ways: in one, the amplitude of each shift about the optimal location was drawn uniformly from the interval between 0 deg and 1 deg visual angle; in the other, the amplitude was drawn from an empirical unimodal frequency distribution taken from a previously published report^61^ (Fig. 3a, Source Data files). With both distributions, the orientation of each shift was drawn uniformly from the interval between 0 deg and 360 deg.

### Summary statistics

Information estimates are reported for individual scenes and summarized by medians to accommodate occasional extreme values in skewed distributions. Uncertainties in estimates were quantified with 95% confidence intervals (CIs) estimated by Efron’s BCa bootstrap method^109^ with 1000 bootstrap replications over scenes.

Except for the outdoor measurements with two observers, fixation distributions were pooled over each observer group to approximate an upper bound on gaze behavior^93^ rather than being based on individual observers^110,111^, as in a previous analysis^53^ where individual detection performance was of interest.

The goodness of fit in linear regressions was summarized by *R*^2^, the proportion of variance accounted for by the explanatory variable after allowing for the degrees of freedom in the regression^112^.

## Data Availability

Hyperspectral images may be downloaded from https://doi.org/10.48420/14877285 and https://doi.org/10.6084/m9.figshare.c.5240420. Fixation data are available on request.
